# SNP rs2057482 in *HIF1A* gene predicts clinical outcome of aggressive hepatocellular carcinoma patients after surgery

**DOI:** 10.1038/srep11846

**Published:** 2015-06-26

**Authors:** Xu Guo, Deyang Li, Yibing Chen, Jiaze An, Kan Wang, Zhuding Xu, Zhinan Chen, Jinliang Xing

**Affiliations:** 1 State Key Laboratory of Cancer Biology, Cell Engineering Research Center & Department of Cell Biology, Fourth Military Medical University, Xi’an, China; 2 Experimental Teaching Center of Basic Medicine, Fourth Military Medical University, Xi’an, China; 3 Department of Hepatobiliary Surgery, Xijing Hospital, Fourth Military Medical University, Xi’an, China; 4 Department of Pain treatment, Tangdu Hospital, The Fourth Military Medical University, Xi’an, China; 5 Department of liver Surgery, Eastern Hepatobiliary Surgery Hospital, The Second Military Medical University, Shanghai, China

## Abstract

Hypoxia-inducible factor 1α (HIF-1α) plays an important role in tumor growth and metastasis. Genetic variations of *HIF1A* gene have been shown to influence the developing risk and prognosis in many types of human malignancies. However, their association with clinical outcomes of hepatocellular carcinoma (HCC) patients remains unclear. To investigate the predictive role of single nucleotide polymorphisms (SNPs) in *HIF1A* gene in HCC patients’ outcomes, we genotyped three functional SNPs (rs2057482, rs1957757 and rs2301113) in *HIF1A* gene and assessed their associations with clinicopathological parameters and prognosis of 492 surgical HCC patients. The patients with variant alleles (CT+TT) of SNP rs2057482 had a significantly lower recurrence risk when compared with patients with the CC genotype. In stratified analysis, the protective effect of rs2057482 CT+TT genotype was more evident in patients with adverse strata, compared with patients with favorable strata. Additionally, strong joint predictive effect between rs2057482 genotypes and AFP level, stage or differentiation were observed. Functional assay also indicated the significant effect of rs2057482 on gene expression. In conclusion, SNP rs2057482 in *HIF1A* gene is significantly associated with clinical outcomes of Chinese HCC patients after surgery, especially in those with aggressive status, which warrants further validation in other patient populations.

Hepatocellular carcinoma (HCC) is the fourth most common cancer and third leading cause of cancer death worldwide^1^. The prognosis of post-operational HCC patients is still poor due to the high recurrence rate. Several factors, such as tumor size, number of tumors, cell differentiation, venous invasion and inflammatory degree, are predictors for prognosis in HCC patients. Despite these substantial factors used in the clinical prediction of HCC prognosis, long-term outcome remains unfavorable for HCC^2^,^3^,^4^,^5^. Hence, it is urgently needed to identify new biomarkers for more effective prognosis prediction and to subsequently improve therapeutic benefit for HCC patients.

Hypoxia inducible factor-1 (HIF-1), a transcription factor, consists of two sub-units –HIF-1α and HIF-1β, both of which belong to the basic loop-helix Per-Aryl hydrocarbon nuclear translocator-Sim (PAS) protein family^6^. HIF-1 coordinates the response to hypoxia in normal and tumor tissues, which allows the cell to adapt and survive in hostile environment. It immortalizes tumor cells by inducing the expression of key genes involved in cancer biological processes, including angiogenesis, glycolysis, invasion, and metastasis^7^.

A series of studies have reported that the overexpression of HIF-1α is associated with an aggressive phenotype and the increased mortality in many cancer types, including HCC^8^,^9^. Moreover, the disease-free survival (RFS) time of HCC patients with high HIF-1α expression was significantly shorter than that of those with low expression^10^. HIF-1α has also been reported to be associated with poor prognosis in several types of other cancers, including non-small cell lung cancer^11^, colorectal cancer^12^, and neuroendocrine breast cancer^13^. Therefore, HIF-1α may be an interesting candidate as a novel prognostic marker.

Single-nucleotide polymorphisms (SNPs) are attractive biomarkers for translational studies due to its easy-to-detect from blood samples^14^. A number of SNPs associated with tumor development and progression have been identified in the *HIF1A* gene. For example, two SNPs, C1772T (rs11549465) and the G1790A (rs11549467) have been found to be correlated with the risk of colorectal cancer^15^,^16^. Another study has demonstrated an association between *HIF1A* c*191T>C (rs2057482) and the risk of rectal cancer^17^. HIF-1α regulates the epithelial-mesenchymal transition (EMT) that is one of the crucial mechanisms to cause early stage of tumor metastasis^18^. One study has reported that the presence of the variant allele A for G1790A is associated with disease-relapse and shorter disease-free survival in oral squamous cell carcinoma^19^. In HCC study, only one report has showed that *HIF1A* SNP G1790A was an important susceptibility factor^20^. However, the association between SNPs of *HIF1A* gene and survivals of HCC remains to be determined. Given the important role of HIF-1α in the progression of HCC, it is plausible that polymorphisms of *HIF1A* may affect the biological behavior and prognosis of HCC. In this study, we selected 3 functional SNPs in the *HIF1A* gene and evaluated their associations with survival in a Chinese cohort of 492 patients diagnosed with HCC.

## Methods

### Study population

Between January 2009 and January 2012, a total of 492 Han Chinese patients with primary HCC were recruited from Xijing Hospital, Fourth Military Medical University (FMMU) in Xi’an and Eastern Hepatobiliary Surgery Hospital, Secondary Military Medical University (SMMU) in Shanghai, China. There was no previous history of other cancers for all patients. All patients received surgery within 2 months after diagnosis and no patient received anticancer treatment before surgery.

Demographic data were collected through in-person interviews at the time of initial visit or follow-up in the clinics, medical chart review, or consultation with the treating physicians by trained clinical research specialists. The follow-up information was updated at 6-month intervals through onsite interviews, direct calling, or medical chart review. The latest follow-up in this study was carried out in January 2013. For each participant, 5 ml of venous blood was collected and used for genomic DNA extraction using the E.Z.N.A. Blood DNA Midi Kit (Omega Bio-Tek, Norcross, GA, USA) in the laboratory.

### SNP selection and genotyping

SNPs in *HIF1A* gene were selected using a set of web-based SNP selection tools (http://snpinfo.niehs.nih.gov/snpinfo/snpfunc.htm) according to the previous description^21^. Briefly, SNPs with minor allele frequency (MAF) <5% in Han Chinese population (CHB) were excluded. Potential functional SNPs were identified to meet the following criteria: SNPs in miRNA binding sites of 3’ untranslated region (UTR), SNPs in the transcription factor binding site of the 5’ flanking region (2000 bp upstream from the transcript start site), SNPs in splice sites and non-synonymous SNPs in exons. And we also included the SNPs which are associated with other diseases in previous studies. Finally, we identified 3 SNPs in *HIF1A* gene, including 1 SNP in the 3’-UTR (rs2057482) and 2 SNPs in intron region (rs1957757 and rs2301113) which have been previously reported to be associated with disease susceptibility^22^,^23^. All selected SNPs were genotyped by iPLEX genotyping system (Sequenom, San Diego, CA, USA). Internal quality controls and negative controls were used to ensure genotyping accuracy, and 5 samples were randomly selected and genotyped in duplicate with 100% concordance. Call rate for genotyping ranged between 99.0% and 99.4%.

### Functional Assay

The luciferase reporter assay was used to assess functional effects of rs2057482 located in the 3’UTR of *HIF1A* gene. First, 53-bp double-stranded oligonucleotides carrying either wild type or variant genotype of SNP rs2057482 were synthesized and cloned into the pMIR-REPORT vector. Human HCC cell lines SMMC-7721 and human embryonic kidney cell line HEK-293T (American Type Culture Collection), in which two microRNAs (miR-199a–5p and miR-340) were identified to be positively expressed by using TaqMan miRNA expression assay (Applied Biosystems, USA) as previously described^24^, were cotransfected with either pMIR-rs2057482-C or pMIR-rs2057482-T (200 ng/well) with or without anti-miR-199a-5p (Applied Biosystems, USA) and the internal control plasmid pRLTK (Promega) (20 ng/well) in a 24-well plate with 2 × 10^5^ cells per well. After 48 hours, the cells were harvested to determine luciferase activity using a dual-luciferase reporter assay system kit (Promega) with a luminometer (Tecan, Mannedorf, Switzerland).

To further evaluate the effect of SNP rs2057482 genotypes on *HIF1A* mRNA expression, total RNA was prepared from 40 HCC tissue samples (20 with CC genotypes and 20 with CT/TT genotypes of rs2057482) using commercial RNA extraction kit (Omega Bio-Tek, Norcross, GA) according to the manufacturer’s instructions. Then, cDNA were synthesized using PrimeScript RT reagent kit (Takara, Japan). The real-time quantitative reverse transcription PCR was performed as previously described^25^ using following *HIF1A* primers: forward, 5’-CGCAAGTCCTCAAAGCACA-3’; reverse, 5’-TCAGTGGTGGCAGTGG TAGT–3’^26^.

### Statistical analysis

For each SNP, three genetic model (dominant, additive and recessive models) were used for analysis. Overall survival (OS) was defined as the time from surgery to death of HCC. RFS was defined as the time from surgery to the first date of recurrence. Hazard ratios (HRs) and 95% confidence intervals (CIs) were estimated by the Cox proportional hazard model after adjusting for age, gender, HBsAg status, AFP level, tumor differentiation, TNM stage, and treatment after surgery. The different genotype groups of significant SNPs were plotted by adjusted survival curve. Statistical significance was set at a level of 0.05 and all analyses were done using the SPSS software package (version 19.0, SPSS, Inc.).

### Ethical statement

The study was approved by the Institutional Review Boards of FMMU and SMMU and informed written consent was obtained from each patient. The experiment was conducted according to the Declaration of Helsinki.

## Results

### Distribution of patients’ characteristics and prognosis analysis

The distribution of patients’ characteristics was summarized in Supplementary Table 1. Among all 492 patients, 185 (37.6%) had received adjuvant transcatheter arterial chemoembolization (TACE) treatment. During the median follow-up of 21.8 months (ranging from 1.6 to 48.3), 179 (36.4%) patients died of HCC, and 295 (60.0%) patients developed recurrence. There were 6 patients who died from other causes and thus were treated as censored data in survival analysis.

Multivariate Cox regression analyses showed that there were significant higher death and recurrence risks in patients with tumor stage III and IV (HR = 3.06 and 2.26, respectively), poor differentiation (HR = 2.40 and 1.82, respectively) and high level of serum AFP (HR = 2.08 and 1.48, respectively) when compared with those patients with tumor stage I and II, good/mediate differentiation and low level of serum AFP, respectively. Patients who received adjuvant TACE after surgery showed a significantly decreased risks of death and recurrence (HR = 0.62 and 0.56, respectively) when compared with those who were treated by surgery alone (Table 1).

### Association of *HIF1A* SNPs with clinical outcome in HCC patients

Cox regression analyses were used to access the associations of *HIF1A* genotypes with HCC survival in three genetic models (Table 2). Our data showed that SNP rs2057482 was significantly associated with RFS of HCC patients under dominant model. Compared to patients with the CC genotype, those with variant alleles (CT and TT genotypes) had a significantly lower recurrence risk (HR = 0.76, 95%CI: 0.59–0.97, *P* = 0.028). Adjusted survival curve showed a significant difference in RFS between patients with the CC genotype of SNP rs2057482 and those with variant alleles (*P* = 0.028)(Supplementary Figure 1). The median RFS was 14.2 months in patients with the CC genotype and 16.9 months in patients with CT/TT genotypes. In addition, SNP rs1957757 was significantly associated with OS of HCC patients under recessive model. Due to very small number of patients carrying genotype TT, we did not perform further analysis for this SNP.

### Stratified analysis on association of rs2057482 with outcomes in HCC patients

**Figure 1:**
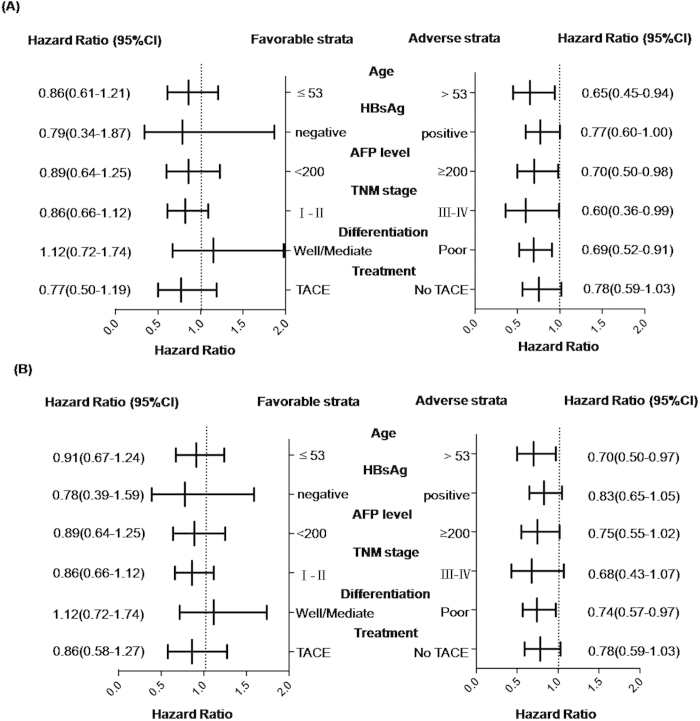
Stratified analysis on association of rs2057482 with outcomes in HCC patients. The HR in (**A**) and (**B**) were indicated for OS and RFS respectively, both figures were stratified by favorable and adverse strata.

The associations between genotypes of rs2057482 and HCC survival were further evaluated in stratified analysis by age, HBV status, AFP level, TNM stage, differentiation and treatment. As shown in Fig. 1A, significant protective effects of CT/TT genotypes on RFS were almost observed in adverse groups with a range from 0.60 to 0.78 of HR. For details, the significant decreased recurrence risk conferred by SNP rs2057482 variant-containing genotypes (CT/TT) was observed in older patients (HR: 0.65; 95%CI: 0.45–0.94), in patients with positive serum HBsAg (HR: 0.77; 95%CI: 0.60–1.00), AFP >200 μg/L (HR: 0.70; 95%CI: 0.50–0.98), clinical stage III-IV (HR: 0.60; 95%CI: 0.36–0.99) and poor differentiation (HR: 0.69; 95%CI: 0.52–0.91). Similar results were also observed in patients without adjuvant TACE treatment although only borderline significance was reached (HR: 0.78; 95%CI: 0.59–1.03; *P* = 0.063). In addition, we found that there was a similar trend in our stratified analysis for OS, also indicating more obvious protective effects of CT/TT genotypes in adverse groups with a range from 0.68 to 0.83 of HR (Fig. 1B).

### Joint effect between SNP rs2057482 and AFP level, stage or differentiation on RFS

To further evaluate the joint effect of rs2057482 and clinical elements (AFP level, stage, differentiation) representing status of tumor progression on HCC RFS, a joint analysis was carried out (Table 3). The data showed that there was a statistically significant interaction between the genotypes of rs2057482 and AFP level, stage or differentiation on RFS (*P* for interaction <0.001 for all of them). Compared to patients with the CT/TT genotypes and a low level of serum AFP, those with CC genotype and a high level of serum AFP had a significantly higher recurrence risk (HR = 1.97, 95%CI: 1.36–2.85, *P* < 0.001). The similar results were observed in stage III/IV patients with CC genotype (HR = 3.17, 95%CI: 2.15–4.68, *P* < 0.001), and in poor differentiation patients with CC genotype (HR = 1.97, 95%CI: 1.28–3.03, *P* < 0.001) when compared to corresponding reference group.

### Functional effects of rs2057482 on gene expression

**Figure 2:**
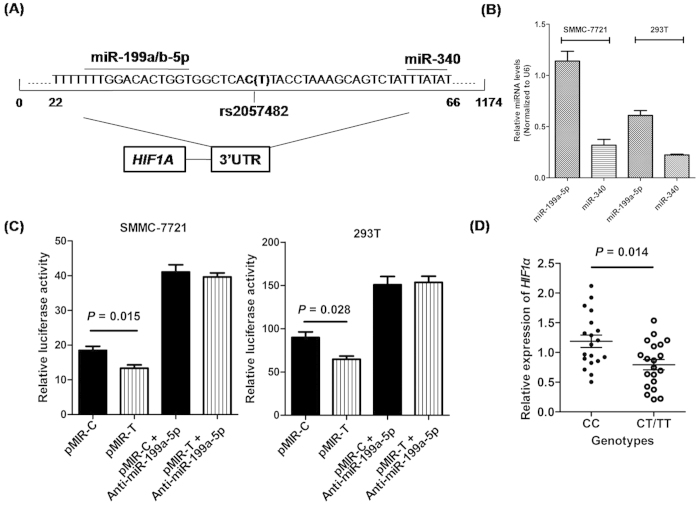
Functional effect of SNP rs2057482 genotypes on gene expression by the luciferase reporter assay. (**A**) The sequence including rs2057482 in 3’UTR of *HIF1A* gene. (**B**) and (**C**) The effects of SNP rs2057482 genotypes on the expression of *HIF1A* gene in SMMC-7721 and HEK-293T cells. Recombinant vector (pMIR-rs2057482-C or pMIR-rs2057482-T) and pRL-TK were co-transfected into 7721 and 293T cells. Data are expressed as Mean ± standard deviation (SD). *T* student test was used to examine the statistical difference.

Bioinformatics analysis (http://www.microrna.org/microrna/home.do) showed that rs2057482 is close to two predicted microRNA binding sites (hsa-miR-199a/b-5p and hsa-miR-340) (Fig. 2A). Moreover, we confirmed the expression of miR-199a-5p and miR-340 in two cell lines, examined with a relatively higher expression level of miR-199a-5p (Fig. 2B). To determine whether the genotypes of SNP rs2057482 in the 3’UTR of *HIF1A* gene could alter the gene expression, SMMC-7721 and HEK-293T cells were transfected with luciferase reporter plasmids containing 3’UTR of the *HIF1A* gene with either wild-type (CC) or variant (TT) genotype of SNP rs2057482 (Fig. 2C). Our results indicated that SNP rs2057482 had a significant influence on the normalized luciferase activity in all transfected cells. Cells transfected with plasmids carrying variant genotype (TT) of SNP rs2057482 exhibited a significant reduction of normalized luciferase activity when compared with cells transfected with plasmids carrying wild-type genotype (CC). We also investigated the effect of anti-miR-199a-5p on the luciferase activity of reporter plasmids. Our results indicated that anti-miR-199a-5p significantly increased the luciferase activity of two UTR constructs (p-MIR-C and p-MIR-T) in both cell lines and obliterated the differences of luciferase activity between two reporter plasmids. We further evaluated the effect of rs2057482 genotypes on mRNA expression of *HIF1A* in 40 HCC tissues (20 with CC genotype and 20 with CT/TT genotypes) using qRT-PCR and found that mRNA expression level of *HIF1A* was significantly lower in patients carrying CT/TT genotypes of rs2057482 than those in patients carrying CC genotype (*P* = 0.014) (Fig. 2D).

## Discussion

In the present study, we examined whether genetic polymorphisms in the *HIF1A* gene are associated with survival in a cohort of 492 resected HCC patients. The most important finding is that CT/TT genotypes of SNP rs2057482 in *HIF1A* 3’UTR region are significantly associated with better HCC RFS when compared with CC genotypes, and functional assay indicated that genotypes of SNP rs2057482 had a significant influence on *HIF1A* mRNA expression in tissues and cell lines. Additionally, the prognostic effect of rs2057482 was more predominant for adverse groups of patients. Furthermore, a significant interaction of gene-clinical characteristics was observed in joint analysis. To the best of our knowledge, this is the first study to report that *HIF1A* gene polymorphisms may serve as an independent prognostic marker for HCC patients with surgical treatment. Once validated, *HIF1A* SNP rs2057482 may be used in combination with traditional clinical prognosis factors for decision-making of HCC treatment.

A few studies have focused on the association between the polymorphisms in *HIF1A* gene and cancer risk or prognosis. Two SNPs, rs11549465 and rs11549467, have been reported to be associated with risk of several cancers^15^,^27^. Another study has observed that the presence of the variant allele A for G1790A is related to disease-relapse and shorter disease-free survival in early stages of oral squamous cell carcinoma^19^. However, we excluded these two SNPs in our study because the minor allele frequencies of them are less than 5% in CHB population. Our study demonstrated that CT/TT genotypes of rs2057482 were significantly associated with better prognosis of HCC patients, suggesting the critical function of HIF-1α in HCC.

A previous study has analyzed the expression of HIF-1α by immunohistochemical staining in 179 tumor specimens and showed that HIF-1α is over-expressed in 13 of 19 tumor types compared with the respective normal tissues, including colon, breast, gastric, lung, skin, ovarian, pancreatic, prostate, and renal carcinomas^8^. Subsequent studies have found that the expression of HIF-1α affects the survival of cancer patients^28^,^29^. A meta-analysis which included 953 HCC patients has suggested that HIF-1α overexpression is correlated with poor prognosis^30^. In mechanism, HIF-1α heterodimerizes with HIF-1β and regulates the expression of downstream target genes. Those HIF-1α-regulated target molecules are commonly demonstrated to modulate tumor migration, invasion, and metastatic property^31^. The SNP rs2057482 is located in the 3’ UTR region of *HIF1A* gene. Bioinformatics analysis (http://www.microrna.org/microrna/home.do) showed that rs2057482 is close to two predicted microRNA binding sites (hsa-miR-199a/b-5p and hsa-miR-340)^32^. Therefore, it is biologically plausible that such a variation at this position may have an effect on mRNA stability and lead to altered binding activity to microRNAs, which might regulate gene expression by mRNA cleavage or translational repression^33^. Our functional assay indicated the potential impact of rs2057482 on the post-transcriptional regulation of *HIF1A* gene by miR-199a-5p. Consistently, SL Fu *et al.*^34^ have also evaluated the *HIF1A* mRNA expression in 17 cervical cancer tissues with genotype data of SNP rs2057482 and found similar result, indicating that the tissues carrying CT/TT genotypes of rs2057482 had significantly decreased *HIF1A* mRNA expression levels when compared to those with CC genotype. Furthermore, we found that the significant or borderline significant protective effects of CT/TT genotypes of rs2057482 on OS and RFS were almost only observed in adverse strata patients, but not in favorable strata patients. Many previous studies have indicated that SNPs affect clinical outcomes more evidently in specific cancer patient subgroups. Wu *et al.* have reported that genetic variations in cell cycle pathway genes exhibit more significant effect on the survival of patients with stages III/IV NSCLC and microRNA-related genetic variants is more remarkably associated with clinical outcomes in early-stage NSCLC patients^35^,^36^. We also observed the strong interactions between SNP rs2057482 and stage, differentiation or AFP levels on prognosis prediction, which suggests that there might be potential modulating roles of these clinical elements representing tumor progression in biological functions of SNP rs2057482. These epidemiological evidences may help us to establish a potential strategy for HCC survival prediction and personalized medicine, but the underlying mechanism needs further investigation.

To conclude, this is the first evidence suggesting that a polymorphism in *HIF1A* gene (rs2057482) has significant impact on clinical outcome in Chinese HCC patients, especially among patients with advantage stage or some other aggressive clinical status. This finding has potential clinical significance in assisting the development of personalized medicine strategies.

## Additional Information

**How to cite this article**: Guo, X. *et al.* SNP rs2057482 in *HIF1A* gene predicts clinical outcome of aggressive hepatocellular carcinoma patients after surgery. *Sci. Rep.* **5**, 11846; doi: 10.1038/srep11846 (2015).

## Supplementary Material

Supplementary Information

## Figures and Tables

**Table 1:** Distribution of HCC patients’ characteristics and prognosis analysis.

**Variable**	**All patients, n (%) n = 492**	**OS**			**RFS**		
		**Death, n (%)n = 179**	**HR^a^ (95% CI)**	***P*-value**	**Recurrence, n (%)n = 295**	**HR^a^ (95% CI)**	***P*-value**
Gender							
Female	67(13.6)	20(11.2)	Ref.		34(11.5)	Ref.	
Male	425(86.4)	159(88.8)	**1.80(1.11–2.91)**	**0.017**	261(88.5)	**1.59(1.10–2.30)**	**0.013**
Age							
<53	253(51.4)	93(52.0)	Ref.		160(54.2)	Ref.	
≥53	239(48.6)	86(48.0)	1.19(0.88–1.61)	0.267	135(45.8)	1.00(0.79–1.27)	0.972
HBsAg							
Negetive	46(9.3)	16(8.9)	Ref.		27(9.2)	Ref.	
Positve	446(90.7)	163(91.1)	1.02(0.60–1.75)	0.940	268(90.8)	1.11(0.74–1.68)	0.615
Serum AFP							
<200	264(53.7)	68(38.0)	Ref.		138(46.8)	Ref.	
≥200	228(46.3)	111(62.0)	**2.08(1.51–2.86)**	**<0.001**	157(53.2)	**1.48(1.16–1.89)**	**0.002**
Differentiation							
I+II	147(29.9)	28(15.6)	Ref.		65(22.0)	Ref.	
III+IV	344(70.1)	151(84.4)	**2.40(1.58–3.65)**	**<0.001**	230(78.0)	**1.82(1.36–2.43)**	**<0.001**
TNM stage							
I+II	394(80.1)	114(63.7)	Ref.		215(72.9)	Ref.	
III+IV	98(19.9)	65(26.3)	**3.06(2.22–4.22)**	**<0.001**	80(27.1)	**2.26(1.72–2.98)**	**<0.001**
Treatment							
Surgery	307(62.4)	114(63.7)	Ref.		193(65.4)	Ref.	
Genotype	185(37.6)	65(36.3)	**0.62(0.45–0.85)**	**0.003**	102(34.6)	**0.56(0.43–0.71)**	**<0.001**

Abbreviations: CI, confidence interval; HR, hazard ratio.

^a^Adjusted for gender, age, HBsAg, AFP level, clinical stage, differentiation and treatment after surgery, where appropriate.

^b^Significant P values (<0.05).

**Table 2:** Association of *HIF1A* SNPs with clinical outcome of HCC patients.

**SNP**	**Genotype**	**OS**			**RFS**		
		**Death/total**^a^	**HR**^b^ **(95% CI)**	***P*****-value**^c^	**Recurrence/total**^a^	**HR**^a^ **(95% CI)**	***P*****-value**^b^
rs2057482	CC	113/302	Ref.		187/302	Ref.	
	CT	61/172	0.81(0.59–1.11)	0.192	96/172	0.74(0.58–0.95)	0.020
	TT	5/13	0.95(0.36–2.49)	0.914	9/13	1.09(0.54–2.17)	0.814
	Additive		0.85(0.64–1.13)	0.256		0.81(0.65–1.02)	0.070
	Dominant		0.82(0.60–1.12)	0.203		0.76(0.59–0.97)	0.028
	Recessive		1.05(0.41–2.72)	0.917		1.24(0.63–2.45)	0.537
rs1957757	CC	162/454	Ref.		273/454	Ref.	
	CT	11/24	0.76(0.39–1.50)	0.433	13/24	0.70(0.38–1.29)	0.252
	TT	5/11	3.16(1.26–7.94)	0.014	8/11	1.75(0.86–3.59)	0.124
	Additive		1.24(0.81–1.91)	0.315		1.07(0.77–1.50)	0.675
	Dominant		1.07(0.61–1.87)	0.819	21/35	0.95(0.59–1.52)	0.815
	Recessive		3.17(1.26–7.97)	0.014		1.77(0.86–3.62)	0.120
rs2301113	AA	70/188	Ref.		117/188	Ref.	
	AC	84/228	0.88(0.64–1.22)	0.444	138/228	0.89(0.70–1.14)	0.366
	CC	23/73	0.68(0.42–1.05)	0.120	38/73	0.71(0.49–1.03)	0.074
	Additive		0.84(0.67–1.05)	0.124		0.86(0.72–1.02)	0.076
	Dominant		0.83(0.61–1.13)	0.244	176/301	0.85(0.67–1.07)	0.172
	Recessive		0.73(0.46–1.15)	0.173		0.76(0.54–1.08)	0.120

Abbreviations: CI, confidence interval; HR, hazard ratio; Ref., reference.

^a^Due to genotyping fail, sample size of individual SNPs may not add up to total.

^b^Adjusted by gender, age, HBV, AFPlevel, stage, Differentiation, treatment.

^c^Significant *P* values (P < 0.05).

**Table 3:** Joint effect of rs2057482 genotypes and AFP level, stage, differentiation on RFS.

**variables**	**Recurrence/Total** a	**HR(95% CI)**^b^	***P*****-value**^c^
TT/CT + low level AFP	46/95	Ref.	
CC + low level AFP	89/164	1.24(0.87–1.78)	0.241
TT/CT + high level AFP	59/90	1.43(0.96–2.13)	0.082
CC + high level AFP	98/138	**1.97(1.36–2.85)**	**<0.001**
*P* for interaction			**<0.001**
			
TT/CT + stage I + II	73/143	Ref.	
CC + stage I + II	139/246	1.24(0.93–1.66)	0.139
TT/CT + stage III + IV	32/42	**2.08(1.36–3.18)**	**<0.001**
CC + stage III + IV	48/56	**3.17(2.15–4.68)**	**<0.001**
*P* for interaction			**<0.001**
			
TT/CT + high/mediate differentiation	25/53	Ref.	
CC + high/mediate differentiation	38/91	0.87(0.52–1.44)	0.580
TT/CT + poor differentiation	79/131	1.33(0.84–2.10)	0.229
CC + poor differentiation	149/211	**1.97(1.28–3.03)**	**0.002**
*P* for interaction			**<0.001**

Abbreviations: CI, confidence interval; HR, hazard ratio; Ref., reference.

^a^Due to genotyping fail, sample size of individual SNPs may not add up to total.

^b^Adjusted by gender, age, HBV, AFPlevel, stage, differentiation, treatment.

^c^Significant *P* values (<0.05).
