# Supplementary material for: SNP rs2057482 in HIF1A gene predicts clinical outcome of aggressive hepatocellular carcinoma patients after surgery
Source: Sci Rep. 2015 Jun 26;5:11846. doi: 10.1038/srep11846 (PMC4481773; doi:10.1038/srep11846)

SNP rs2057482 in *HIF1A* gene predicts clinical outcome of aggressive hepatocellular carcinoma patients after surgery

Xu Guo[1#], Deyang Li[1#], Yibing Chen[2], Jiaze An[3], Kan Wang[4] Zhuding Xu[5], Zhinan Chen[1*], Jinliang Xing[1,2*]

Figure legend

Figure S1. Survival curves of HCC patients by SNP rs2057482. (A) Overall survival. (B) Recurrence-free survival.

Figure S1. Survival curve of rs2057482 in HCC.

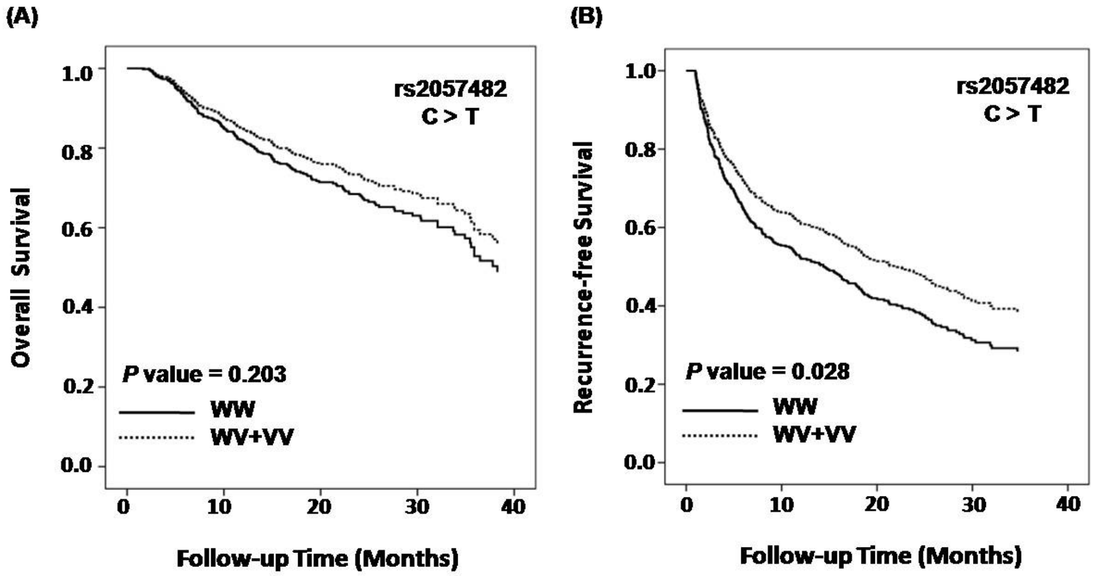

Supplement: Supplementary Information [file srep11846-s1.pdf]
